# Textiloma mimicking Crohn's disease in its features: A case report

**DOI:** 10.1016/j.amsu.2021.01.016

**Published:** 2021-01-16

**Authors:** Abdul Rahman Hammadieh, Mhd Belal Alsabek, Sara Rustom

**Affiliations:** a Department of Surgery, Almouwasat University Hospital, Damascus University, Faculty of Medicine, Damascus, Syria; b Department of Surgery, Syrian Private University, Faculty of Medicine, Damascus, Syria

**Keywords:** Gossypiboma, Textiloma, Gauzoma, Crohn's disease, Enteroenteric fistula

## Abstract

**Introduction and importance:**

Textiloma is a retained surgical item such as a sponge or gauze that is unintentionally left in the surgical field after the wound closure. Here, we present the first reported surgical gauze that penetrated the intestine, made a duodenal-ceca fistula and then stuck far away in the ileum. Mechanical obstruction didn't appear clinically or even in radiological investigations because of the fistula which provided the intestinal continuity.

**Case presentation:**

We report a 34-year-old man with a previous abdominal interventions complained of cramping, frequent vomiting and presence of undigested food in stool. The frequency of the bowel movement increased recently. Endoscopies, radiological investigations and pathological findings figure out a duodenal-ceca fistula with nonspecific inflammatory tissues in the intestinal biopsy. When we performed the abdomen surgery, retained gauze in the ileum was taken out and the duodenal-ceca fistula was fixed.

**Clinical discussion:**

Gauze or sponge that is forgotten in the surgical field called gossypiboma, textiloma, gauzoma or cottonoid. It could present with various complaints; as an acute or chronic problem, clear or ambiguous symptoms. It could reside in a space; extend across a gap, migrate through a tissue, or even make a fistula between lumina like in our case.

**Conclusion:**

Textiloma could change pre-operative diagnosis, intra-operative techniques, postoperative follow-up plan and prognosis. This is the first report proves its ability to mimic inflammatory diseases that penetrate two different lumina and perform fistula. So it should be written in the list of any differential diagnosis when the patient has a previous procedure or surgery.

## Highlights

• Textiloma couldn't be always diagnosed preoperatively.
• It could give unrelated symptoms to its mass effect or contaminated problems.
• It could mimic Crohn's disease clinically, radiologically and even pathologically.
• It is a deferential diagnosis for every patient who has a previous procedure or surgery.
• Exploring the whole entire abdomen is necessary before performing the techniques.

## 1 Introduction

This work has been reported in line with the SCARE 2020 criteria [1]. Textiloma is derived from textile (a woven fabric), gossypiboma is derived from Latin gossypium (the genus of cotton plants) and gauzoma is taken from surgical gauze [2]. All these three items are retained surgical items such as sponge, instrument, tool or device that is accidentally left in the patient at the completion of a surgery or other procedure [3]. Retained gauze is mostly seen in the abdomen, but it has been reported in the spine, limb, and thorax [[4], [5], [6], [7]]. Textiloma could appear in many manifestations such as abscess, acute abdominal pain and mass effect.

In our manuscript, we are going to present textiloma in which the gauze totally migrated into the intestine and left a duodenal-cecum fistula at the point of entrance; this fistula hid the symptoms of mechanical obstruction that happened in the ileum where the gauze was eventually stuck.

## 2 Case presentation

A 34-year-old male referred to our university clinic with a complaint of cramping and presence of undigested food in stool. The frequency of the bowel movement increased recently. Frequent vomiting was also developed. The patient denied bloody stool, weight loss, fever or chills.

**Fig. 1:**
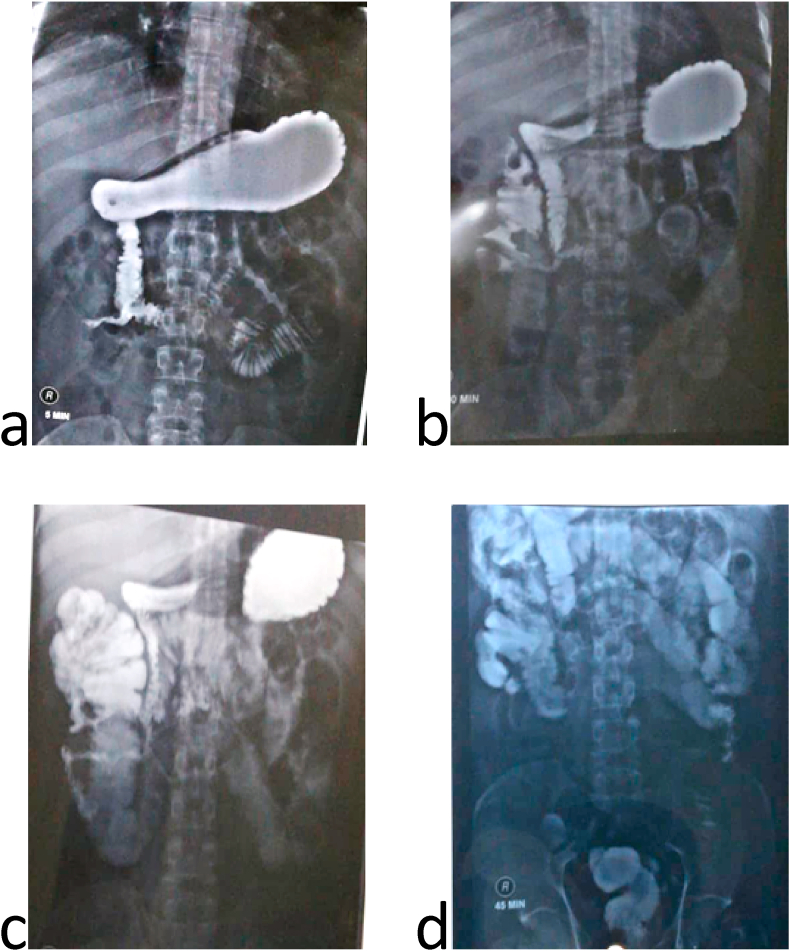
Upper gastrointestinal series: a) &b) A fistula between the 2nd segment of duodenum and cecum, c) mild distention and wall thickness of ileal loops, d) rapid and early contrast filling in the rectum (because of the fistula).

**Fig. 2:**
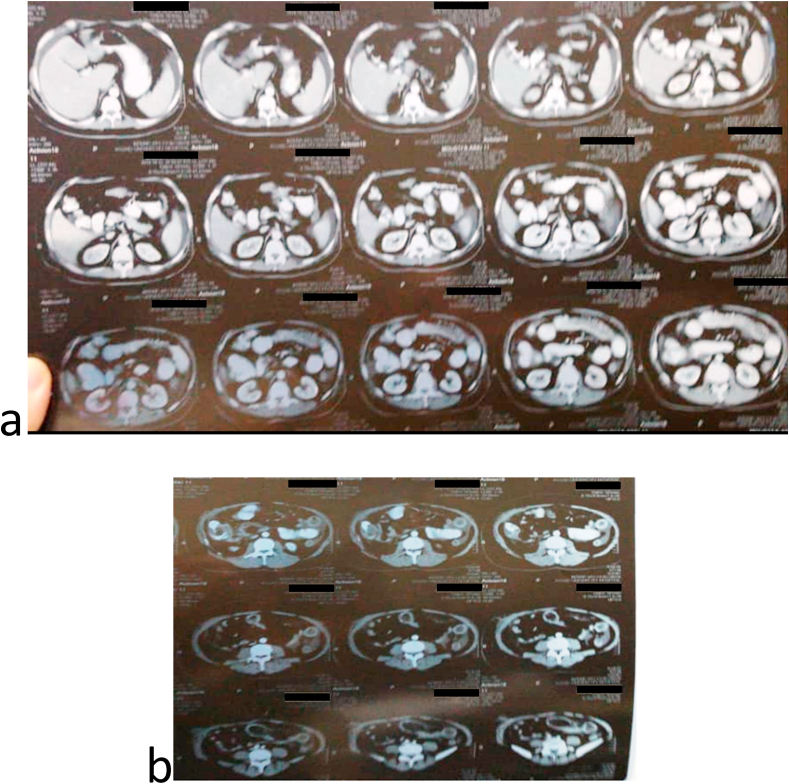
Contrast abdominal computed tomography scan: a) A fistula between the 2nd segment of duodenum and cecum, cecal wall thickening, mild enlarged pericecal lymph nodes, b) small bowel wall thickening in the left upper quadrant and change in the density of the fat around.

**Fig. 3:**
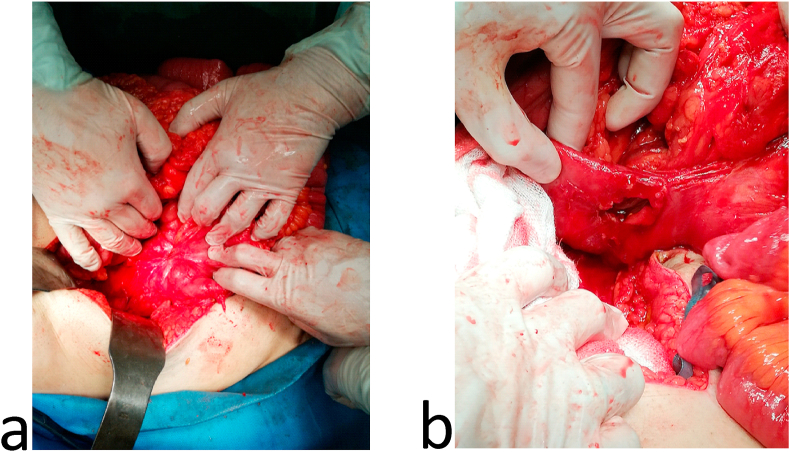
a) Fistula between the 2nd segment of duodenum and cecum; b) fistuloectomy.

**Fig. 4:**
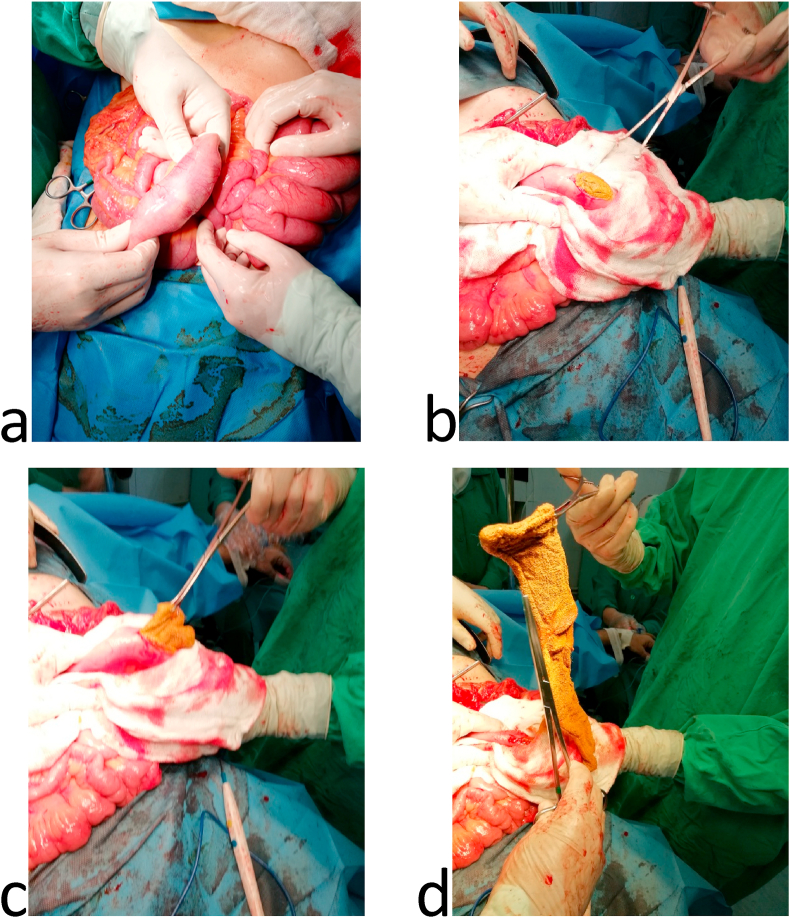
a), b), c) & d) Intraluminal gauze was detected and exerted from the ileum.

In the past, the patient had open appendectomy five months ago, laparoscopic cholecystectomy seven months ago and open varicocelectomy twenty years ago. Past medical history, family medical history, psychosocial history and drug were unremarkable. His routine labs were unremarkable, and the abdominal ultrasound had no specific findings. Upper gastrointestinal series proved an enterocolic fistula [Fig. 1]. Abdominal computed topography (CT) scan displayed the fistula between the 2nd segment of the duodenum and the cecum. It also showed a small bowel wall thickening [Fig. 2]. The patient underwent colonoscopy which was normal except a (≤2 cm) polyp in the cecum. It was resected and its histology was a hyperplastic. Upper gastrointestinal endoscopy found erythematous in the third part of the duodenum. Biopsies were taken from this area and the results showed non-specific inflammatory features. These were the only notable preoperative investigations which suggested Crohn's disease. Surgical decision was made to improve the patient's compliant. Intraoperatively, the duodenal-cecum fistula was found [Fig. 3]. Before we fixed it, we explored an intraluminal mass of the ileum. It was gauze [Fig. 4]. We exerted it, resected the fistula of intestine and then restored the intestine continuity. Postoperative monitoring was unremarkable. The patient's diet went back normal gradually in days. Follow-up for one year showed there were no complications and the procedure relieved the symptoms.

## 3 Discussion

Textiloma is reported in one out of 1000–15000 intra-abdominal operations [7]. The risk factors for retained instruments and sponges after surgery include emergent surgery, high body mass index, busy surgical fields and unplanned changes in procedures [8]. Textiloma should be considered as a differential diagnosis of any mechanical obstruction in patients who underwent abdominal surgery [2]. The radiological diagnosis of textiloma is easily made by plain abdominal radiography, when a radio-opaque marker is seen. However, these markers could disintegrate or fragment over years after the surgery [9]. Foreign bodies may completely migrate into the ileum without any apparent opening in the intestinal wall, but they usually cannot pass the ileocecal valve and cause complete obstruction [10]. However, the mechanical obstruction didn't act in our case because of the duodenal-cecum fistula that had made by the gauze. That fistula which appeared on the contrast series and CT scan directed the physicians to put Crohn's disease as an initial diagnosis. Crohn's disease used to be the first accused disease can result in fistula formation in the gastrointestinal tract [11]. But we learned from our case that occasionally the absolute diagnosis couldn't be reached till an exploratory surgery is done. The surgical technique to fix the fistula didn't perform before the whole entire abdomen was explored. The complete mechanical obstruction was silent in the ileum; leaving it unintentionally would definitely add further malpractice, increase the suffering of the patient postoperatively and return him to the operating room urgently.

## 4 Conclusion

Gauze that is forgotten in the surgical field could present later with various symptoms in days, months or even years. It is not uncommon to make the diagnosis by an exploratory surgery. Textiloma could change any pre-operative diagnosis, so it should be in the list of any differential diagnosis when the patient has a previous procedure or surgery.

## Ethical approval

Informed consent was taken for this case report. Our study ethical aspects were reviewed and approved by Damascus University deanship, Damascus, Syria.

## Sources of funding

No funding was received for this study.

## Consent

Written informed consent was obtained from the patient for publication of this case report and accompanying images. A copy of the written consent is available for review by the Editor-in-Chief of this journal on request.

## Author contribution

All the authors made an equal contribution to the creation of the presented clinical case.

*Abdul Rahman Hammadieh*: the assistant professor and the chief surgeon who prepared the patient and run the operation. ahammadieh@yahoo.com.

*Mhd Belal Alsabek*: attending Surgeon, corresponding author, collected the data, reviewed the PubMed Library and wrote the manuscript. drsabekb@gmail.com.

*Sara Rustom*: undergraduate medical student who participated in writing the manuscript. cu.ty.sara@hotmail.com.

## Registration of research studies

1. Name of the registry: This case report is not a first time of reporting new device or surgical technique. So I would not need a Research Registry unique identifying number (UIN).
2. Unique Identifying number or registration ID:
3. Hyperlink to your specific registration (must be publicly accessible and will be checked):

It is a case report not a research.

## Guarantor

Mhd Belal Alsabek.

## Provenance and peer review

Not commissioned, externally peer-reviewed.

## Declaration of competing interest

The authors declare that there is no conflict of interests regarding the publication of this paper.

## References

[1] Agha R.A., Franchi T., Sohrabi C., Mathew G., for the Scare Group (2020). The SCARE 2020 guideline: updating consensus surgical CAse REport (SCARE) guidelines. Int. J. Surg..

[2] Rajagopal A., Martin J. (2002 Jan). Gossypiboma "a surgeon's legacy": report of a case and review of the literature. Dis. Colon Rectum.

[3] Copeland A.W. (2019). Retained surgical sponge (gossypiboma) and other retained surgical items: prevention and management. https://www.uptodate.com/contents/retained-surgical-sponge-gossypiboma-and-other-retained-surgical-items-prevention-and-management.

[4] Okten A.I., Adam M., Gezercan Y. (2006). Textiloma: a case of foreign body mimicking a spinal mass. Eur. Spine J..

[5] Sadeghifar A.R., Saeed A.R. (2013 Sep). Infected textiloma, 35 Years after the operation for femur fracture, an extremely rare occurrence. Arch Bone Jt Surg.

[6] Nobre L.F., Marchiori E., May F., Carrão A.D., Zanetti G., Machado D.M. (2010 Jan). Thoracic textilomas after myocardial revascularisation: typical CT findings. Br. J. Radiol..

[7] Dane C., Yayla M., Dane B. (2006). A foreign body (gossypiboma) in pregnancy: first report of a case. Gynecol. Surg..

[8] Gawande A.A., Studdert D.M., Orav E.J., Brennan T.A., Zinner M.J. (2003). Risk factors for retained instruments and sponges after surgery. N. Engl. J. Med..

[9] Zbar A.P., Agrawal A., Saeedi I.T., Utidjian M.R.A. (1998). Gossypiboma revisited: a case report and review of the literature. J. R. Coll. Surg. Edinb..

[10] Manzella A., Filho P.B., Albuquerque E., Farias F., Kaercher J. (2009 Dec). Imaging of gossypibomas: pictorial review. AJR Am. J. Roentgenol..

[11] Zuiki T., Meguro Y., Kumano H. (2014). Successful management of a colo-duodenal fistula in a patient with crohn's disease using a double lumen gastro-jejunostomy tube. Case Reports in Gastroenterology.

